# Balance between matrix metalloproteinases (MMP) and tissue inhibitors of metalloproteinases (TIMP) in the cervical mucus plug estimated by determination of free non-complexed TIMP

**DOI:** 10.1186/1477-7827-6-45

**Published:** 2008-09-30

**Authors:** Naja Becher, Merete Hein, Niels Uldbjerg, Carl Christian Danielsen

**Affiliations:** 1 Department of Obstetrics and Gynecology, Århus University Hospital, Skejby, DK-8200 Aarhus N, Denmark; 2 Department of Connective Tissue Biology, Institute of Anatomy, University of Aarhus, DK-8000 Aarhus C, Denmark

## Abstract

**Background:**

The cervical mucus plug (CMP) is a semi-solid structure with antibacterial properties positioned in the cervical canal during pregnancy. The CMP contains high concentrations of matrix metalloproteinase 8 and 9 (MMP-8, MMP-9) and tissue inhibitor of metalloproteinase 1 (TIMP-1). This indicates a potential to degrade extracellular matrix components depending on the balance between free non-complexed inhibitors and active enzymes.

**Methods:**

Thirty-two CMPs collected during active labor at term were analyzed. Twelve CMPs were separated into a cellular and an extracellular/fluid phase and analyzed by gelatin and reverse zymography to reveal MMP and TIMP location. Twenty samples were homogenized, extracted and studied by the TIMP activity assay based on gelatin zymography. Enzyme-linked immunosorbent assay (ELISA) was used to determine TIMP-1, MMP-8 and MMP-9 protein concentrations, and gelatin and reverse zymography used to identify gelatinases and TIMPs, respectively. The Western blotting technique was applied for semi-quantification of alpha2-macroglobulin. An ELISA activity assay was used to detect MMP-8 and MMP-9 activity.

**Results:**

ProMMP-2, proMMP-9, TIMP-1 and TIMP-2 were almost exclusively located in the fluid phase compared to the cellular phase of the CMP. All the extracted samples contained MMP-8, MMP-9, TIMP-1, TIMP-2 and alpha2-macroglobulin. Free non-complexed TIMP was detected in all the samples analyzed by the TIMP activity assay and was associated with TIMP-1 protein (R = 0.71, p < 0.001) and with the TIMP/MMP molar ratio (1.7 (1.1–2.5) (mean (95% confidence interval)) (R = 0.65, p = 0.002). The ELISA activity assay showed no activity from MMP-8 or MMP-9.

**Conclusion:**

Due to their extracellular location, potential proteolytic activity from neutrophil-derived MMPs in the CMP could exert a biological impact on cervical dilatation and fetal membrane rupture at term. The functional TIMP activity assay, revealing excess non-complexed TIMP, and a molar inhibitor/enzyme ratio above unity, indicate that refined MMP control prevents CMP-originated proteolytic activity in the surrounding tissue.

## Background

The cervical mucus plug (CMP) constitutes a strong barrier between the sterile uterine environment and the microbe-rich vagina and protects the fetus from ascending infection throughout pregnancy [1-3]. We now know that the CMP not only has antibacterial properties, but also contains high concentrations of the matrix metalloproteinases (MMPs) gelatinase A (MMP-2), gelatinase B (MMP-9) and neutrophil collagenase (MMP-8) together with their specific inhibitors, the tissue inhibitors of metalloproteinases, TIMP-1 and TIMP-2 [4].

There exists an extensive quantity of literature on the subject of MMP and TIMP in human reproduction. We and others have linked MMP protein in the CMP [4], the cervix [5], the fetal membranes [6,7] and in amniotic fluid [8,9] to proteolytic activity in connection with term and preterm birth. It is tempting to explain cervical dilatation and membrane rupture solely on the basis of MMP protein detection and presumed MMP activity. But the functional balance between matrix-degrading enzymes and their inhibitors [10,11] in the CMP and the heterogeneity of cervical matrix biology [12,13], are fundamental to our understanding of complicated multi-faceted processes like term and preterm birth.

While working with MMP and TIMP in the cervical mucus plug, an important question arises: does the detection of MMP protein imply proteolytic activity? To answer this question, we have conducted the present study where biological enzyme availability, i.e. enzyme and inhibitor location in the CMP cellular or extracellular phases, will be analyzed and discussed. Furthermore we will describe the presence of free non-complexed MMP-inhibitors as an indicator of the functional inhibitor-enzyme balance using a *TIMP activity assay*.

Several traditional methods, such as enzyme-linked immunosorbent assay (ELISA), reverse zymography and western blotting, can detect TIMP protein [14]. Common to all these methods is that they, together with the free TIMP fraction, identify TIMP already complexed with MMPs. Determination of the fraction of non-complexed TIMP describes the physiological impact of MMP presence and for this purpose we have developed the *TIMP activity assay *which is able to estimate free TIMP.

The aim of the present work was to elucidate the proteolytic capacity of the MMPs previously detected in the CMP. This capacity depends upon three factors: whether or not the enzymes are released from the cells, their activation status and finally the degree of inhibition by endogenous inhibitors present in the fluid phase. The aim was approached by 1) localization of MMP and TIMP proteins to cell phase or fluid phase, 2) determination of the molar ratio of MMP inhibitors/MMPs, 3) quantification of active MMPs and 4) determination of non-complexed TIMP by development of a *TIMP activity assay*.

## Methods

### Materials

The 32 intact CMPs included in this study were shed spontaneously or manually retrieved during vaginal exploration (active labor, cervical dilatation from 2–10 cm). The CMPs were either directly suspended in PBS (1:100) for determination of TIMP and MMP distribution between the cellular and the extracellular phases (n = 12, suspension group) or frozen for later extraction (n = 20, extraction group). The women were healthy and their mean age was 29 (range 16–36) years; they had all been through a normal pregnancy and delivered vaginally at term (gestational week 37–42). The Central Denmark Region Committee on Biomedical Research Ethics approved the project and informed consent was obtained from each patient. MMP-1EA (MMP-1 with no enzymatic activity but retained TIMP-binding capacity) and APMA-activated MMP-2 were kindly donated by Dr. Yoshifumi Itoh, The Kennedy Institute of Rheumatology, Imperial College, London.

### CMP suspension

The samples (n = 12) were diluted directly after collection without prior freezing. They were dissolved in PBS to a final 1:100 dilution using a mantoux syringe to ensure gentle suspension with no cell damage. After centrifugation (10 min, 500 g, 4°C), the supernatant (fluid phase) and the precipitate (cell phase) were separated and the cell phase was re-suspended to the original 1:100 dilution. Cells were trypanblue dyed (0.5% w/w, Bie & Berntsen A/S, Rødovre, DK) and counted (Bürker-Türk cell counter chamber). To ensure that the suspension procedure did not cause cell damage and protein leakage, it was performed on two buffy coats containing fresh donor blood leukocyte fractions supplied by the blood bank. Samples were kept at -80°C until analysis.

### CMP extraction

Samples (n = 20) were extracted as previously described [4]. Briefly, mixed pulverized CMP was extracted in 50 mM Tris-HCl, 10 mM CaCl_2_, 0.05% Brij 35 and 1 mM PMSF, pH 7.4 two times overnight at 4°C and once at 60°C (4 min). The three supernatants were pooled (final 1:100 dilution) and kept at -80°C until analysis.

### Gelatin zymography

Gelatin zymography was performed on CMP extractions, CMP suspensions and the two buffy coats. For the CMP suspensions and the buffy coats, the purpose was to analyze gelatinase activity in the fluid and the cell phases. From the identical fluid phase and cell phase dilutions (1:100), 0.5 μl was loaded on a 10% gel. After electrophoresis performed as previously described [4], the proMMP-2 and proMMP-9 bands were scanned using a Shimadzu Cromato Scanner (CS-930, Shimadzu Corporation, Kyoto, Japan) for semi-quantification of gelatinolytic activity in the two phases.

### Reverse zymography

Sixteen μl of fluid and cell phase (CMP and buffy coat suspensions) or CMP extracts was mixed 1:1 with electrophoresis sample buffer and incubated for 15 min at 37°C, and the gels (13%) were loaded with 30 μl of this mixture. TIMPs were identified by comparison with the following standards: TIMP-1 (4.0 ng/lane, Biogenesis, Pole, UK) and TIMP-2 (4.0 ng/lane, Sigma-Aldrich).

### TIMP activity assay

**Figure 1:**
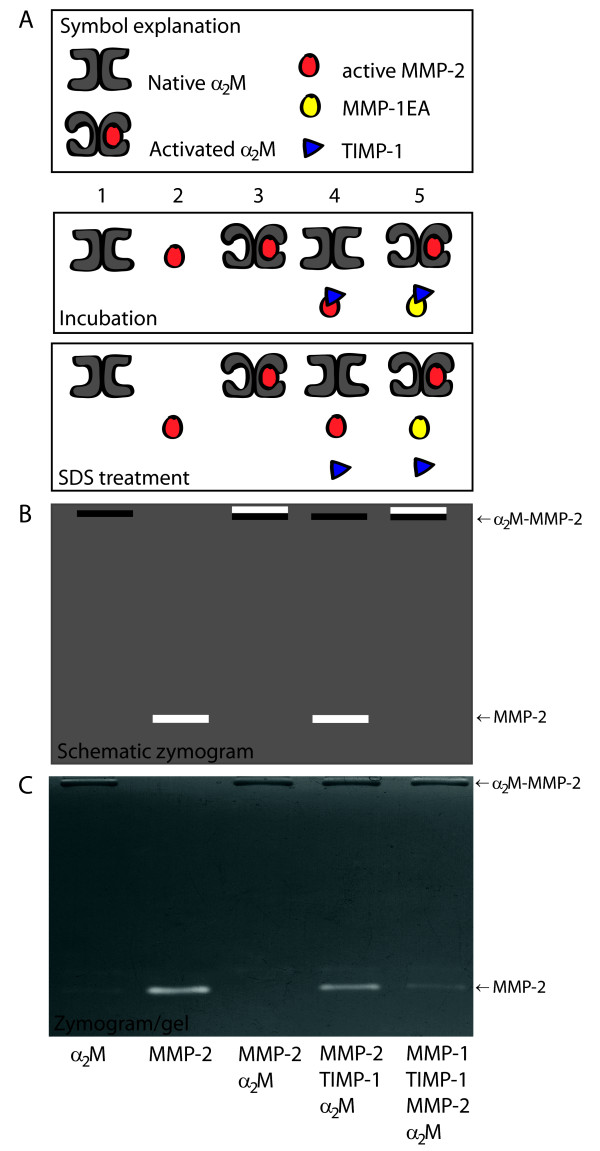
**Schematic illustration of the *TIMP activity assay***. The steps involved in *TIMP activity assay *during incubation, SDS treatment (inactivates MMPs, their reactivation is accomplished by SDS removal after electrophoresis) and zymography, respectively. (A): Symbol explanation and schematic illustration, (B): schematic zymogram and (C): a true zymogram. In *lane 1*, α_2_M, represented in a monomeric form (180 kDa), is visible as a high molecular weight protein band at the gel top. In *lane 2*, activity from active MMP-2 is visible as a lysis band at 64 kDa. *Lane 3 *illustrates the basic principle of the assay: active MMP-2 cleaves the α_2_M bait region during incubation, is entrapped and stays covalently entrapped after SDS treatment. This is evident by a lysis band at the top of the zymogram together with α_2_M. In *lane 4*, MMP-2 is pre-incubated with TIMP before the adding of α_2_M. During this pre-incubation TIMP will bind to MMP-2 and inhibit enzyme activity. When α_2_M is thereafter added, MMP-2 cannot cleave the bait region. The following SDS treatment disassociates TIMP from MMP-2, and MMP-2 reappears on the zymogram as a 64 kDa lysis band. In *lane 5*, TIMP-1 is pre-incubated with MMP-1EA (TIMP-binding capacity but without enzymatic activity) which results in TIMP-1 capture. When MMP-2 and α_2_M are thereafter simultaneously added, MMP-2 is active and free to cleave the α_2_M bait region resulting in entrapment. SDS disassociates the TIMP/MMP-1EA complex but the α_2_M-MMP-2 complex stays intact. On the zymogram only the α_2_M-MMP-2 complex is visible at the gel top.

This assay is built on the premise that active, but not inhibited, MMP-2 is entrapped by α_2_-macroglobulin (α_2_M) [15]. By mixing CMP extract, APMA-activated MMP-2, and α_2_M prior to gelatin zymography, it is possible to conclude on the presence of free inhibitor in the sample since the MMP-2 (64 kDa) lysis band intensity is brought to reflect the concentration of free inhibitor in the CMP extracts. Figure 1 illustrates the basic principle of the assay.

The CMP extracts (n = 20) were all scanned for the presence of free MMP inhibitors by α_2_M MMP-2 entrapment. For each CMP, the wells were loaded with sample mixture corresponding to sample A) CMP (2 μl) + MMP-2 (10 pg), and sample B) CMP (2 μl) + MMP-2 (10 pg) + α_2_M (0.5 μg). Sample A versus sample B demonstrate the change in MMP-2 (64 kDa) lysis activity when α_2_M is added to a mixture of CMP and active MMP-2. Sample B) was prepared by simultaneous mixing of MMP-2 and α_2_M (a gift from Lars Sottrup-Jensen, Department of Molecular Biology, University of Aarhus, Denmark) into the CMP extract. This mixture was incubated for 30 min at 37°C. Before electrophoresis on gelatin mini-gels (7.5%) performed as previously described [4], all samples were diluted 1:1 with electrophoresis sample buffer (4% SDS, 20% glycerol, 0.1% bromophenol blue, 125 mM Tris-HCl, pH 6.8) and incubated for 15 min at 37°C. After electrophoresis and staining, the MMP-2 (64 kDa) lysis bands (representing the fraction of 10 pg MMP-2 that the inhibitors in CMP were able to bind) were scanned and the lysis intensity was quantified and compared to the intensity of the standard containing 10 pg of active MMP-2 alone.

Eight extracts were titrated at declining volumes (from 2.0 μl to 0.0625 μl) with constant MMP-2 (25 pg) and α_2_M (0.5 μg) concentrations. Moreover, to test whether the free inhibitor was indeed TIMP, other aliquots of these 8 samples (4 μl) were pre-incubated 15 min at room temperature with MMP-1EA (see Materials above) in excess (4 ng) in order to capture all free TIMP possibly present in the CMPs. Then, MMP-2 (20 pg) and α_2_M (1 μg) were added simultaneously (total volume 10 μl). Half of the sample volume (mixed 1:1 with sample buffer) was loaded onto the gels and electrophoresed as described above. In this test experiment, all the free TIMP present in the CMP sample is captured by MMP-1EA and MMP-2 is not expected to give lysis band at 64 kDa (i.e. all MMP-2 is entrapped by α_2_M) unless the CMP also contains non-TIMP inhibitors. Control experiments to test the functionality of the reagents comprised four wells: 1) α_2_M (0.5 μg), 2) MMP-2 (10 or 25 pg), 3) MMP-2 + α_2_M, and, finally, 4) MMP-2, TIMP-1 or TIMP-2 (50 pg) and α_2_M in combination.

### Enzyme-linked immunosorbent assay (ELISA)

TIMP-1, MMP-8 and MMP-9 concentrations in the CMP extracts were determined in duplicate by means of a commercially available ELISA-kits based on a two-site sandwich format (GE Healthcare, Buckinghamshire, UK, product number RPN2611, RPN2619 and RPN2614, respectively). We have previously validated this assay system for use on CMP extracts [4].

*ELISA activity assay *(GE Healthcare) was performed on CMP extracts according to the manufacturer's instructions. Active MMP-8 (product number RPN2635) was measured in a range between 0.75–24.0 ng/ml. The sensitivity was 1.2 ng/ml, the inter-assay coefficient of variation (CV) was less than 19.0% and the intra-assay CV less than 13.2%. Active MMP-9 (product number RPN2634) was measured in a range between 0.5–16.0 ng/ml. The sensitivity was 0.125 ng/ml, the inter-assay CV was less than 43% and intra-assay CV was less than 21.7%.

### Western blotting

Sixteen μl of CMP extracts was mixed 1:1 with electrophoresis sample buffer and incubated for 15 min at 37°C. A 6% gel was loaded with 30 μl of sample mixture together with α_2_M standard (10 ng and 50 ng). Following electrophoresis under non-reducing conditions, proteins were blotted to a PVDF membrane using a wet transfer system (Bio-Rad Laboratories, California, USA) with cooling in 25 mM Tris, 192 mM glycine, 0.05% SDS, pH 8.3 as transfer buffer. Membranes were blocked with 0.5% Tween 20/PBS for 0.5 h and then probed overnight at 37°C with primary antibody (rabbit anti-human α_2_M (A033, DAKO, Glostrup, DK) diluted 1:500 in 0.5% skim milk powder/0.05% Tween 20/PBS). For detection, we used alkaline phosphatase-conjugated goat anti-rabbit antibody (D487, DAKO) as secondary antibody and 5-bromo-4-chloro-3-indolyl phosphate/nitroblue tetrazolium as substrate. The α_2_M content was semi-quantitated in duplicate according to the standards using ImageJ software (NIH, USA).

### Statistics

To obtain a normal distribution and an equal variance, the data were log-transformed when required. Linear regression analysis was used to investigate the relationship between the result of the TIMP *activity assay *and 1) molar TIMP-1 concentration, 2) the molar inhibitor/enzyme ratios as well as the relationship between concentrations of MMP-8 and MMP-9. Protein concentration and molar ratio are presented as mean or geometric mean (95% confidence interval (CI)). The Linear regression analysis was performed and the confidence intervals calculated using SigmaStat^® ^3.5 (Systat). *P*-values below 0.05 were considered significant.

## Results

CMPs from 32 women in active vaginal delivery were analyzed. The mean weight was 5.15 g (range 0.94–11.7 g). Twelve samples were directly diluted and separated into a fluid and a cell phase for determination of TIMP and MMP presence in the two phases. The remaining 20 samples were homogenized to obtain a representative measure of total MMP, TIMP and α_2_M protein.

### TIMP and MMP location

**Figure 2:**
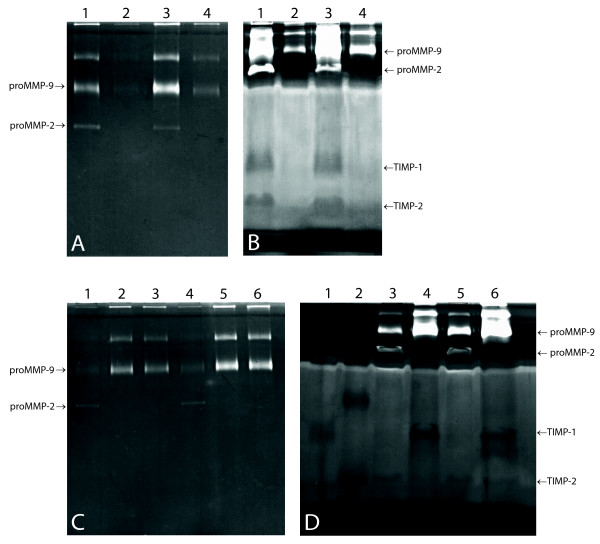
**MMP and TIMP location**. (A): gelatin zymography (10% gel) and (B): reverse zymography (13% gel) on the same two CMP suspensions; *lanes 1 and 3*: CMP fluid phases; *lanes 2 and 4*: CMP cell phases. Both enzymes and inhibitors are almost exclusively present in the fluid phases. (C): gelatin zymography performed on the two buffy coats; *lanes 1 and 4*: fluid phase, *lanes 2–3 and 5–6*: duplex of cellular phase. (D): Reverse zymography. *Lane 1*: TIMP-1 standard (4 ng, 28.5 kDa), *lane 2*: TIMP-2 standard (4 ng, 21 kDa), *lanes 3 and 5*: fluid phase. *Lanes 4 and 6*: cellular phase. For the buffy coats, TIMPs and MMPs are present in the cellular phase.

We used reverse zymography and gelatin zymography to determine the presence of TIMP and MMP in the CMP fluid or cellular phases. ProMMP-2, proMMP-9, TIMP-1 and TIMP-2 were preferably located in the fluid phase (Figure 2A and 2B). On the gelatin zymograms, the ratio of proMMP-2 + proMMP-9 between the fluid phase and the cell phase was estimated by scanning of lysis band intensities and found to be 2.6 (1.4–3.8) (mean (95% CI)). Control experiments with two buffy coats showed MMP and TIMP to be located in the cell phases, which confirmed that the suspension procedure did not damage the cells with consequential MMP release (Figure 2C and 2D). All the 12 suspended plugs contained numerous cells (8.05 × 10^3 ^(3.52 × 10^3^-18.4 × 10^3^) cells/mg CMP) (geometric mean (95% CI)). The vast majority was recognized as leukocytes, but epithelial cells and possibly macrophages were also present. Five samples contained a few vital cells, maybe due to fresh blood contamination; otherwise all the cells were non-vital.

### Inhibitor-enzyme balance

**Figure 3:**
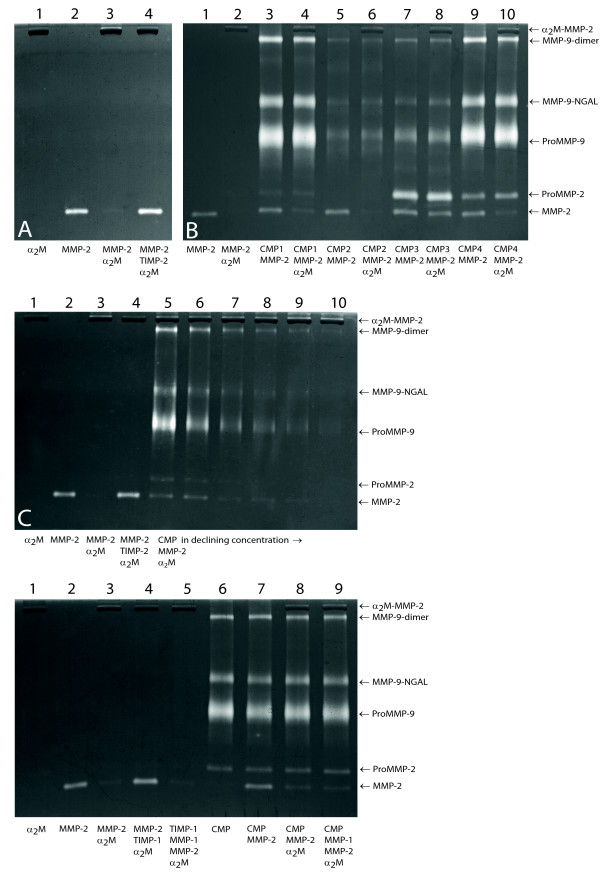
**Functional balance as illustrated by the *TIMP activity assay***. Panel (A) shows the standards as follows: *lane 1*: α_2_M (0.5 μg); *lane 2*: active MMP-2 (25 pg); *lane 3*: α_2_M + MMP-2; *lane 4*: MMP-2 + TIMP-2 + α_2_M. (B): TIMP activity assay applied to four different CMPs: *lane 1*: MMP-2 standard (10 pg), *lane 2*: MMP-2 + α_2_M. *Lanes 3, 5, 7 and 9*: CMP (2 μl) + MMP-2 (10 pg). *Lanes 4, 6, 8 and 10*: CMP + MMP-2 + α_2_M. MMP-2 (64 kDa) lysis bands suggest that an inhibitor in the sample prevented α_2_M-MMP-2 capture. (C): Titration of one inhibitor-positive plug. *Lane 1–4*: controls. *Lanes 5–10*: MMP-2 (64 kDa) lysis activity declines gradually with declining CMP concentration (2, 1, 0.5, 0.25, 0.125 and 0.0625 μl). (D): MMP-1EA-TIMP capture experiment. *Lane 1–4*: controls. *Lane 5*, MMP-1EA capture control: initial incubation of MMP-1EA (2 ng) and TIMP-1 (50 pg) followed by simultaneous addition of MMP-2 (10 pg) and a 2 M (0.5 μg); MMP-1EA forms a complex with TIMP-1 and allows α_2_M-MMP-2 capture resulting in disappearance of MMP-2 (64 kDa) lysis band. In *lane 6–9*, the MMP-1EA-TIMP capture experiment is applied to a CMP. *Lane 6*: CMP (2 μl); *lane 7*: CMP + MMP-2 (10 pg); *lane 8*: CMP + MMP-2 + α_2_M (0.5 μg); *lane 9*: initially CMP and MMP-1EA (2 ng) followed by MMP-2 and α_2_M. MMP-2 (64 kDa), proMMP-2 (72 kDa), proMMP-9 (92 kDa), MMP-9-NGAL complex (125 kDa), MMP-9 dimer (184 kDa) and the α_2_M-MMP-2 complexes are indicated. The analysis was performed on a 7.5% gel.

In Figure 3A the appearance and the functionality of the reagents used in the *TIMP activity assay *are demonstrated. This assay revealed free inhibitor in all 20 samples as illustrated by varying intensity of MMP-2 (64 kDa) lysis activity after α_2_M incubation (Figure 3B). After running a gel with 8 samples premixed with constant concentrations of active MMP-2 and α_2_M but with declining CMP extract concentrations, the MMP-2 (64 kDa) lysis bands were scanned. Lysis activity declined gradually with declining CMP concentrations, which demonstrated the inhibitory potential of each of the CMPs (Figure 3C). Capture of free TIMP by excess amount of MMP-1EA (Figure 3D) revealed that TIMP was not exclusively responsible for the inhibitor activity: the MMP-2 (64 kDa) lysis band was not completely abolished (Figure 3D, lane 9) and, therefore, not all the free inhibitor had been captured by MMP-1EA.

**Figure 4:**
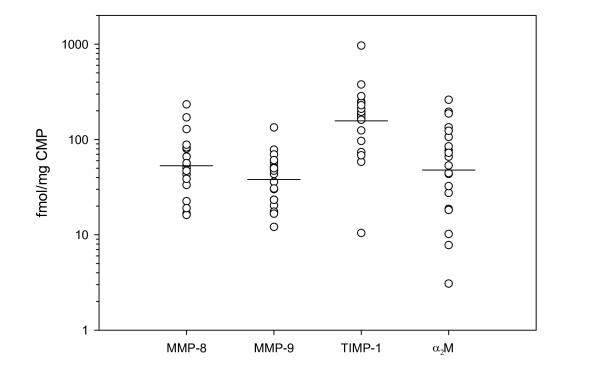
**CMP content of enzymes and inhibitors**. The molar concentrations of CMP MMPs and inhibitors in fmol/mg CMP. Every dot represents a CMP (n = 20) and geometric mean values are indicated. Note the log-scale.

**Figure 5:**
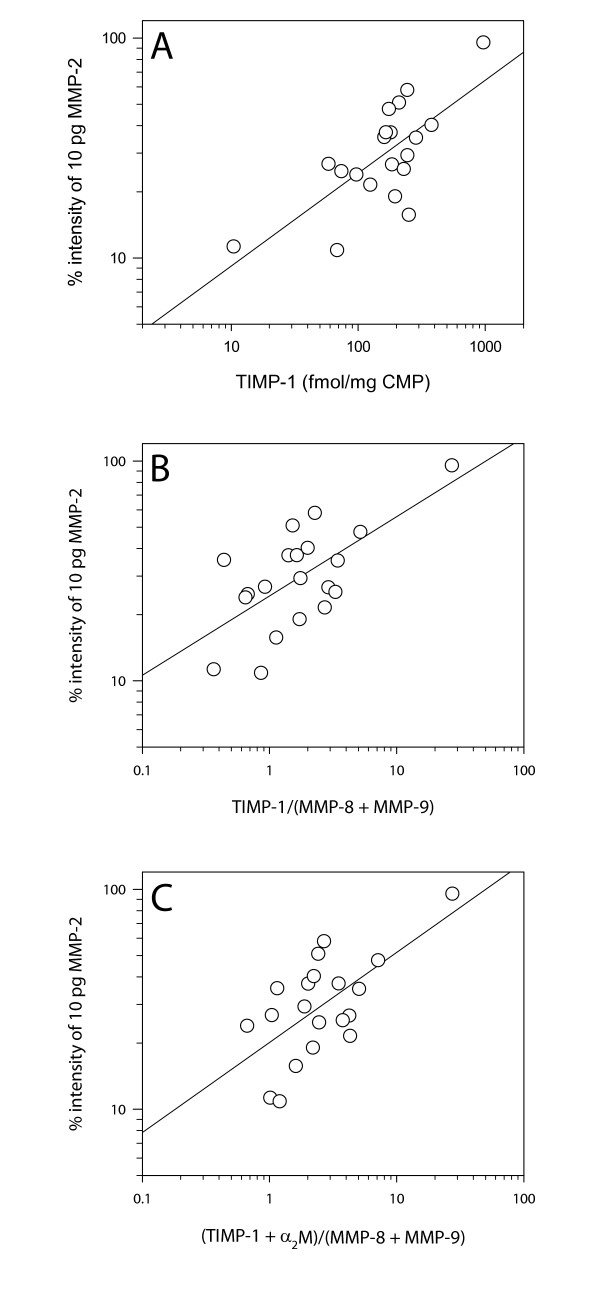
**Regression analysis**. Linear regression analysis of *TIMP activity assay *results versus (A) TIMP-1 molar protein concentration (R = 0.71, p < 0.001), (B) the TIMP-1/(MMP-8 + MMP-9) molar ratio (R = 0.65, p = 0.002) and (C) the (TIMP-1 + α_2_M)/(MMP-8 + MMP-9) molar ratio (R = 0.64, p = 0.002). The MMP-2 (64 kDa) lysis band intensities represent the fraction of 10 pg MMP-2 that the inhibitors in CMP were able to bind in percent of the intensity of the standard containing 10 pg of active MMP-2 alone.

**Figure 6:**
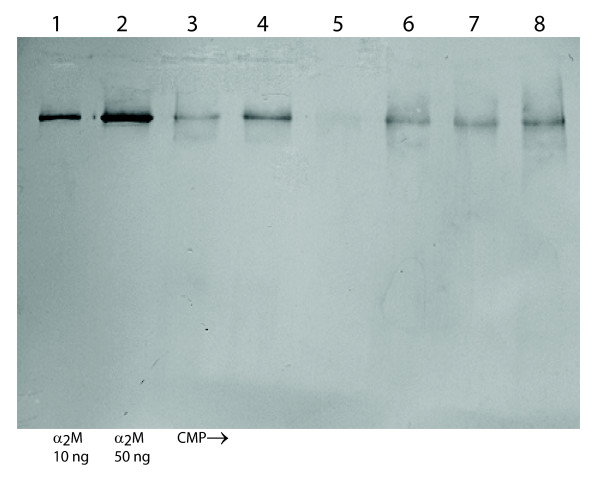
**α_2_-macroglobulin Western blot**. *Lanes 1 and 2 *contain standards, 10 and 50 ng α_2_M (180 kDa monomer), respectively. *Lanes 3–8 *illustrate α_2_M in 6 different CMP extracts.

Nineteen samples contained TIMP-1 concentrations detectable by ELISA (mean 4.71 (3.10–7.16) (95% CI) ng/mg CMP) (molar concentration (fmol/mg CMP) shown in Figure 4). The TIMP-1 concentration varied considerably between the plugs (range < 1.25–29.0 ng/mg CMP). A high TIMP-1 molar concentration was associated with a large free inhibitor capacity as detected by the *TIMP activity assay *(R = 0.71, p < 0.001) (Figure 5A). The molar TIMP-1/(MMP-8 + MMP-9) ratio was above unity in 14 of 20 samples (mean 1.7 (1.1–2.7) (95% CI)) and associated with free inhibitor capacity (R = 0.65, p = 0.002) (Figure 5B). All 20 plugs revealed bands from both TIMP-1 (28.5 kDa) and TIMP-2 (21.5 kDa) on the reverse zymograms corresponding to the standards (not shown). Western blot proved α_2_M presence in all the samples (Figure 6) and the concentrations were semi-quantitatively estimated from band intensity (mean 34.5 (20.0–59.4) (95% CI) ng/mg CMP) (molar concentration (fmol/mg CMP) shown in Figure 4). The molar ratio of total inhibitor (TIMP-1 + α_2_M)/(MMP-8 + MMP-9) was above unity in 18 of 20 samples (mean 2.5 (1.7–3.7) (95% CI)) and associated with free inhibitor capacity (R = 0.64, p = 0.002) (Figure 5C).

ELISA activity assays showed no active MMP-8 or active MMP-9. The mean protein concentration was for MMP-8 3.98 (2.84–5.59) (95% CI) ng/mg CMP and for MMP-9 3.51 (2.65–4.65) (95% CI) ng/mg CMP (molar concentration (fmol/mg CMP) shown in Figure 4). Total MMP-8 protein was directly proportional to total MMP-9 protein (R = 0.85, P < 0.001) (not shown). Gelatin zymography revealed the characteristic lysis bands from MMP-9: bands at 184 kDa (dimer), at 125 kDa (MMP-9/NGAL complex), and at 92 kDa (proMMP-9) in all 32 samples (not shown). Moreover, 16 of 32 samples revealed proMMP-2 lysis bands at 72 kDa, but there were no signs of active MMP-2 in any of the samples.

## Discussion

With a combination of different methods we have tried to assess the proteolytic capacity of the MMPs previously detected in the cervical mucus plug at term of pregnancy. We mainly found both TIMP and MMP in the CMP fluid phase which could mean that they play a role in the biological function of the CMP. If the CMP, when positioned in the cervical canal during the course of pregnancy, can be compared to CMPs collected during active labor at term, the very high CMP MMP concentration might promote premature cervical softening and preterm premature rupture of the membranes (PPROM). The risk of these adverse effects is increased if the enzymes are both present in the fluid phase and represented in their active forms.

Granulocyte counting in mucus from the external cervical os reveals a significant increase in cells during the course of pregnancy with a further increase during active labor [16]. Microscopical observations performed on the mucus plug show cell clusters mainly in the part of the plug facing the vagina, and the predominant cell type observed here is the neutrophil leukocyte [3]. Therefore, the relatively high concentrations of MMP-8 and MMP-9 found in CMPs harvested during cervical dilatation probably derives from neutrophil invasion of the CMP as also evidenced by the CMP neutrophil count performed in this study. Indeed, the concentrations of MMP-8 and MMP-9 were correlated, which signifies a common cellular origin.

If the CMP MMP content primarily results from neutrophil leukocyte degranulation or apoptosis locally in the plug, the enzyme concentration may simply reflect CMP inflammation and antibacterial properties. The great inter-individual variation in enzyme concentrations among the CMPs analyzed could arise from differences in vaginal flora virulence and the consequential CMP antibacterial state of alert. MMP exudation from the dilating cervix undergoing extensive remodeling [17-19] could also contribute to CMP MMP, but to what degree is unknown. Additionally, recruitment of neutrophils caused by labor-induced inflammation may also add to the MMP level that we detect. We suggest that the neutrophil-derived MMPs in the CMP are expressed throughout pregnancy and reflect the main function of the CMP: to prevent preterm birth by protecting the fetus from ascending infection. As opposed to this clearly beneficial impact of neutrophil presence, the MMP content of neutrophil secretory granules may affect the cervix as well as the membranes in case of insufficient inhibition of the activated enzymes.

The *TIMP activity assay *is a most interesting method that builds on the basic principles of 1:1 stoichiometric MMP-TIMP binding and α_2_M active proteinase entrapment, thus detecting free MMP inhibitors. We have applied this principle to the CMP and proved the functional inhibitor-enzyme balance to be in favor of MMP-inhibition: all the CMP extracts analyzed contained free MMP inhibitor in various amounts. In spite of an extracellular location, the CMP MMPs are likely to play a minor role in the extensive cervical remodeling that occurs throughout an active birth process.

No active MMP-8 or MMP-9 could be demonstrated by the ELISA activity assays. The most abundant MMPs in the CMP are not present in their active forms, but either in their pro-forms or complexed with TIMPs. The possibility exists that the concentrations of active MMPs are below detection limit of the kits used since the physiological concentrations of active MMPs in a tissue usually is very low. However, due to excess free TIMP, as demonstrated by the *TIMP activity assay*, this is not likely to be the case when considering the CMP. Should the pro-forms become activated, they will be immediately controlled by the free TIMP fraction. The formation of complexes between TIMPs and MMPs is rapid and strong; only restricted momentary pericellular activity [20,21], and not general proteolytic activity, is possible in a TIMP dominated environment [22,23]. The potential activity of MMP-8 and MMP-9 in the CMP seems to be tightly regulated by MMP-inhibitors which prevent uncontrolled CMP-originated proteolytic activity in the surrounding tissues.

Efforts have been made to quantify the non-complexed free fraction of TIMP-1 in plasma by means of an immunoassay technique [24]. Concerning the CMP, the *TIMP activity assay *supplements immunodetection because it creates an *in vitro *interaction between the endogenous free CMP inhibitor and the added, active MMP-2. A functional reconstruction of highly complex interactions may allow generalization of the results to actual *in vivo *conditions. Control experiments with MMP-1EA capture of CMP TIMP-1 showed that the free inhibitor was not represented by TIMP-1 alone, but the association between the *TIMP activity assay *results and a high TIMP/MMP molar ratio is in favor of TIMP-1 being mainly responsible. A parallel analysis using both techniques would clarify the issue of non-complexed MMP inhibitor in the CMP and reveal if the free inhibitor found here is actually TIMP-1.

On the basis of a molar ratio, we found 1.7 times as much TIMP-1 in the CMP than MMP-8 and MMP-9 together which support the inhibitor superiority detected by the *TIMP activity assay*. When calculating the molar ratio, it must be taken into consideration that only TIMP-1, MMP-8 and MMP-9 were measured. According to our previous studies [4], MMP-8 and MMP-9 are the two most abundant MMPs present in the CMP. We have measured MMP-1 and MMP-7 in CMP extracts and found them to be undetectable. Neither MMP-3 could be detected (casein zymography, unpublished data). MMP-2, on the other hand, is measurable in low concentrations in extracted CMP, but was not included in the present study. However, other MMPs and MT-MMPs not yet characterized in the CMP may also be potential inhibitor targets. The reverse zymography showed TIMP-2 in the CMP samples, but nevertheless the TIMP-2 concentration is unquantifiable with the ELISA technique (levels below kit sensitivity (3.0 ng/ml)). Thus, the molar inhibitor-enzyme ratio calculated on the basis of our results should be taken merely as an indication of an overall tendency towards inhibitor superiority.

The TIMP source may be cervical tissue exudation, endocervical gland secretion or secretion from vital or dead cells already present in the plug. The CMP contains predominantly neutrophil leukocytes but diverging opinions exist regarding their ability to produce TIMP-1. Opdenakke et al. state, that neutrophils are first-line defense leukocytes and that they do not produce gelatinase A (MMP-2) or TIMP-1 [25]. On the other hand, Triebel et al. have succeeded in isolating TIMP-1 from neutrophils, which presumably allow these cells to control their own MMP activity [26]. Concerning the CMP, the most likely option is that TIMP-1 is secreted from the endocervical glands together with the mucus that constitutes the plug.

If CMP TIMP originates from endocervical glands, the regulation of cervical TIMP synthesis and secretion must adjust to the highly individual MMP content to reach the refined enzymatic control that we describe. Such a regulatory mechanism is interesting in the context of preterm birth; if the cervix fails to modify TIMP levels to CMP MMP levels during the course of pregnancy, active proteolytic enzymes may cause cervical softening and/or PPROM leading to preterm delivery. Much effort has been displayed to use different inflammatory markers in cervical secretions to predict preterm labor [27]. A CMP sample is easy to obtain, and with TIMP and MMP preferably located in the fluid phase, these components can be reliably quantified. Future analysis of TIMP and MMP in CMP samples collected during the course of pregnancy may add to our understanding of term labor mechanisms and preterm labor pathophysiology.

## Conclusion

In the present study we found that MMP and TIMP are located in the fluid rather than in the cellular phase of the CMP at term, that the CMP displays molar inhibitor excess, that the CMP contains no active MMP-8 or MMP-9 and finally, that the CMP contains free non-complexed MMP inhibitor which signifies a functional inhibitor superiority.

Our results suggest that the CMP, with neutrophil-derived MMPs available in the fluid phase, has a potential to display matrix degrading properties and therefore could play a role in the cervical dilatation and fetal membrane rupture at term. However, the *TIMP activity assay*, revealing free non-complexed TIMP, and the detection of a molar inhibitor/enzyme ratio above unity, indicate, that inhibitor control hinders CMP-originated proteolytic activity in the surrounding tissue. The regulation and biological importance of the refined CMP inhibitor-enzyme balance described here is yet to be established.

## Competing interests

The authors declare that they have no competing interests.

## Authors' contributions

NB, MH, NU and CCD have participated in the design of the study. The experiments were carried out by NB and CCD. NB, NU and CCD participated in the data analysis. The manuscript was written by NB. MH, NU and CCD assisted in revising it. All authors have read and approved the final manuscript.
